# Genome-Wide Analysis Elucidates the Role of *CONSTANS-like* Genes in Stress Responses of Cotton

**DOI:** 10.3390/ijms19092658

**Published:** 2018-09-07

**Authors:** Wenqiang Qin, Ya Yu, Yuying Jin, Xindong Wang, Ji Liu, Jianping Xi, Zhi Li, Huiqin Li, Ge Zhao, Wei Hu, Chuanjia Chen, Fuguang Li, Zhaoen Yang

**Affiliations:** 1 Xinjiang Research Base, State Key Laboratory of Cotton Biology, Xinjiang Agricultural University, Urumqi 830052, China; qinwenqiang2005@163.com; 2 Institute of Cotton Research, Chinese Academy of Agricultural Sciences, Anyang 455000, China; yuy1025@163.com (Y.Y.); 15757176231@163.com (Y.J.); liujicricaas@163.com (J.L.); xijianping71@126.com (J.X.); li17630615719@163.com (Z.L.); qinqindeshijie@163.com (H.L.); Zhao123abcde.ok@163.com (G.Z.); 18317776546@163.com (W.H.); 3 School of Life Sciences, Central China Normal University, Wuhan 430079, China; wxdnick@hotmail.com

**Keywords:** Gossypium, *CO-like*, segemental duplication, stress response, flowering time

## Abstract

The *CONSTANS (CO)-like* gene family has been well studied for its role in the regulation of plant flowering time. However, their role remains poorly understood in cotton. To better understand the possible roles of *CO-like* in cotton, we performed a comprehensive genome-wide analysis of *CO-like* genes in cotton. Phylogenetic tree analysis showed that *CO-like* genes naturally clustered into three groups. Segmental duplication and whole genome duplication (WGD), which occurred before polyploidy, were important contributors to its expansion within the At (“t” indicates tetraploid) and Dt subgenomes, particularly in Group III. Long-terminal repeat retroelements were identified as the main transposable elements accompanying 18 genes. The genotype of *GhCOL12_Dt* displayed low diversity; it was a candidate involved in domestication. Selection pressure analyses indicated that relaxed purifying selection might have provided the main impetus during the evolution of *CO-like* genes in upland cotton. In addition, the high expression in the torus and calycle indicated that *CO-like* genes might affect flowering. The genes from Group II, and those from Group III involved in segmental duplication or WGD, might play important roles in response to drought and salt stress. Overall, this comprehensive genome-wide study of the *CO-like* gene family would facilitate further detailed studies in cotton.

## 1. Introduction

The *CONSTANS* (*CO*) genes encode a small class of transcription factors, characterised by a CCT (CO, CO-like, TOC1 (TIMING OF CAB EXPRESSION1)) domain near the carboxy terminus. The *CO*-*like* gene family is subdivided into three groups according to the additional B-box domain near the amino terminus. The genes of Groups I, II, and III have two, one and one zinc-finger B-boxes, respectively; moreover, Group III genes contain a diverged B-box [1]. The CCT domain is involved in nuclear localisation of CO proteins, and the B-box plays vital roles in protein–protein interactions [2,3].

In *Arabidopsis*, 17 members of the *CO-like* gene family are divided into three groups. Group I consists of *CO* and *COL1* to *COL5*, Group II consists of *COL6*, *COL7*, *COL8*, and *COL16*, and Group III consists of *COL9* to *COL15* [1]. Increasing numbers of *CO-like* genes have been identified in several plant species, except *Arabidopsis*, owing to the increased availability of genome data [4]. Most studies show that *CO-like* genes play significant roles in the regulation of flowering time in plants. For example, the *Arabidopsis CO* gene play important roles in the photoperiod pathway and regulation of flowering time [5,6], and its ortholog, *Hd1* (*Heading date 1*), in rice shows a similar biological function and acts as a pivotal regulator in the photoperiod pathway [7,8]. 

Furthermore, *AtCOL5* [9], *AtCOL9* [10], and *AtCOL12* [11], and the *OsCOL9* [12], *OsCOL10* [13,14], *OsCOL15* [15], and *OsCOL16* [16] are involved in regulation of flowering time. In addition to the role in flowering time, recent studies have shown that CO-like genes are associated with plant architecture, abiotic stress response, and auxin homeostasis [17,18,19,20,21]. *AtCOL3* is a positive regulator of photomorphogenesis that acts downstream of *COP1* (*Constitutive photomorphogenic 1*) and can promote root growth independent of *COP1* [17]. *AtCOL4* positively regulates abiotic stress tolerance in plants through abscisic acid (ABA)-dependent pathway. In rice, a *CO-like* gene, *Ghd2* (Grain number, plant height, and heading date2), is a negative regulator for drought tolerance, mediated through regulation of senescence in rice [18]. In *Arabidopsis*, *AtCOL7* is involved in the phytochrome B (phyB)-mediated shade avoidance response and enhanced branching number under high red light:far-red light ratio through phyB-mediated light-quality regulation of auxin homeostasis [19]. *AtCOL12* is the substrate of *COP1/SPA* E3 ligase, and the over-expression of *COL12* results in decreased apical dominance and enhanced branching [20]. In rice, *OsCOL9* regulates blast resistance through salicylic acid and ethylene signalling pathways [21].

Cotton is the leading fibre crop worldwide and provides large amounts of raw materials for textile fibres and edible oils [22,23,24]. Few studies on *CO-like* genes in cotton are reported, e.g., ectopic over-expression of cotton *COL1* in *Arabidopsis* rescues the late flowering phenotype of the co-2 mutant [25], but the biological function of most of the *CO-like* genes are unknown in cotton. Therefore, it is important to understand the biological consequences of the activity of the *CO-like* gene family in cotton plants. The completion of the cotton genomes makes it possible to perform a comprehensive genome-wide analysis of *CO-like* genes in cotton [26,27,28,29,30]. Several *CO-like* genes have been reported in monocotyledonous and dicotyledonous plants, but the evolutionary relationships among these genes are poorly understood.

In the present study, we aimed to identify the *CO-like* genes from eight different species and use the sequence information for analysing the relationship between monocotyledonous and dicotyledonous plants. Furthermore, we focused on cotton and comprehensively analysed *CO-like* genes in the allotetraploid upland cotton and two diploid species resembling its wild parents. We performed the identification of gene families, phylogenetic tree analysis, and analysis of segmental duplication, gene structures, domestication selection, chromosome location, and expression patterns.

## 2. Results

### 2.1. Gene Identification and Conserved Domain Retrieval

In total, 21, 18, 38, 11, 16, 15, 13, and 16 genes were confirmed as *CO-like* family members in *G. raimondii*, *G. arboreum*, *G. hirsutum*, cacao, *Populus trichocarpa*, rice, sorghum, and maize, respectively (Table S1). *G. hirsutum* contained the largest number of *CO-like* genes, which was close to that of the two diploid cotton species, *G. arboreum* and *G. raimondii* (Table S2). The number of *CO-like* genes in the diploid cotton *G. arboreum* exceeded that in maize, rice, sorghum, cacao, and poplar. Because *G. raimondii* (DD) has a smaller genome size than *G. arboreum* (AA) and *G. hirsutum* (DD), it has a higher assembly quality than the two latter species. Therefore, firstly we designated the genes from *G. raimondii* as *COL1* to *COL21*, and named the genes from *G. arboreum* (A genome), At subgenome and Dt subgenome of *G. hirsutum* after their orthologs in D genome. The orthologs from *G. raimondii*, At subgenome, and Dt subgenome showed good collinearity with homologous chromosomes (Table S2), suggesting that the *CO-like* genes were conserved in different cotton species during evolution.

### 2.2. Multiple Sequence Alignment of Conserved Domains

The *CO-like* was characterised by two conserved domains. The first one was the CCT, which was located near the carboxy terminus, and the second was a zinc-finger B-box domain located near the amino terminus [1]. To better understand the characteristics of the sequences of *CO-like* genes in cotton, we performed multiple alignments of B-Box and CCT domains. As shown in Figure S1, all genes from upland cotton used in the present study contained the CCT and B-box domain. Nineteen genes contained one CCT domain and two B-boxes (Figure S1A), six genes contained one CCT domain and one B-box domain (Figure S1B), and twelve genes contained one CCT domain, one B-box domain, and one divergent B-box (Figure S1C). Previous reports show that the CO-like genes are divided into three groups based on number and type of B-box. We followed the designation rules and classified the genes in Figure S1A–C into Groups I–III [1]. The sequences of CCT were more conserved within the same groups than among different groups. The sequences of CCT at most of the loci were consensus sequences, and only some loci were divergent. For example, the amino acid residue at 2 (E or V), 9 (R or K), 12 (R or K), 14 (N, K, or T), 18 (E or V), 21 (I or V), 28 (A or S), 32 (M or S), and 41 (A or V) were different. The B-box is a class of zinc fingers, characterised by C-X2-C-X8-C-X7-C-X2-C-X4-H-X8-H, which was identified in *Arabidopsis*, rice and barley. In the present study, we found that B-box in cotton followed the regular pattern mentioned above, suggesting that the B-boxes are highly conserved during the evolution.

### 2.3. Phylogenetic Analysis of CO-like Genes among Typical Dicots and Monocots

**Figure 1:**
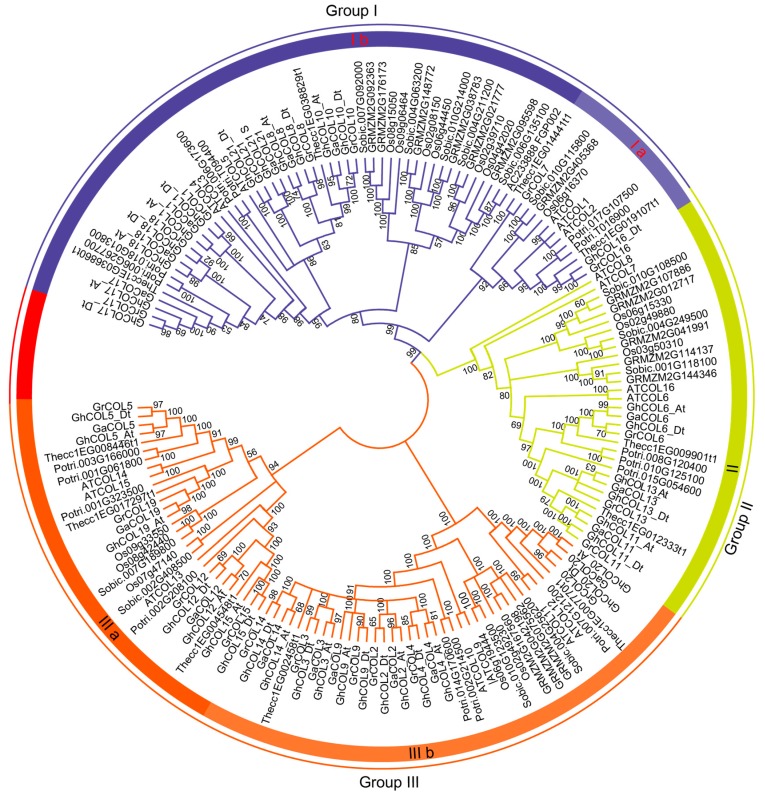
Phylogenetic tree of CO-like genes suggesting that CO-like genes were clustered into three groups. The neighbour-joining (NJ) tree was constructed using MEGA 7.0. The outer circle is marked in purple, green, and orange, which represent Groups I, II and III, respectively. The prefixes At, Os, Potri, GRMZM, Sobic, Thecc, Ga, Gr, and Gh, represent *A. thaliana*, *Populus trichocarpa*, *Oryza sativa*, *Zea mays*, *Sorghum bicolor*, *Theobroma cacao*, *Gossypium arboreum*, *G. raimondii*, and *G. hirsutum*, respectively. “At” and “Dt” indicate the A and D subgenomes in upland cotton, respectively. Bootstrapping was used to check the reliability of the tree, and bootstrapping values larger than 50 are displayed near the nodes.

To understand the relationship among the selected species better, we constructed phylogenetic trees. As shown in Figure 1, the *CO-like* genes were naturally clustered into three groups, designated as I–III. The topology *of* Maximum likelihood (ML) tree (Figure S2) was similar to that in Figure 1 by neighbour-joining (NJ) method, suggesting that the NJ tree was suitable for further analysis. Group III had the largest number of *CO-like* genes, whereas Group II had the smallest number of *CO-like* genes. According to the topology of the tree, Groups I and III have been divided into two sub-groups, termed as a and b, indicating that these two group have experienced expansion, and that their function may have diversified during evolution. The gene numbers from each species in Group II were between two and four, suggesting that this group might not have experienced expansion, and that their biological functions may be very similar.

As shown in Figure 1, the genes from cacao always clustered close to cotton, suggesting that the genes of these two species are closely related (Figure 1). The total number of *CO-like* genes in the diploid *G. raimondii* was twice of that in cacao, and one cacao gene at the most had three orthologs in diploid cotton. For example, in Group III, *Thecc1EG017297t1* only had one ortholog each in the two diploid cotton species, and *Thecc1EG002458t1* corresponded to three orthologs in diploid cotton.

Although the orthologs from three cotton species used in the present study display high sequence similarities, the orthologs from the At subgenome and A genome tended to cluster together. This was similar to the tendency observed among the orthologs from the Dt subgenome and D genome. These similarities suggested that the orthologs from At-A or Dt-D might have a common ancestor.

### 2.4. Gene Expansion and Synteny Analysis

As shown in Figure S3, 36 genes displayed a significant collinearity in the At and Dt subgenomes. Thirty-seven genes were located on 16 chromosomes unevenly, and none of the genes were distributed on 10 chromosomes. Five genes were located on A08 and D08, followed by four on D07/A07, three on D13, two on A01/D01/D13, and one on A05/D05/A09/D09/A12/D12. Only *GhCOL21_S* was located on scaffold4822 because of incomplete sequencing (Table S2 and Figure S3). 

**Figure 2:**
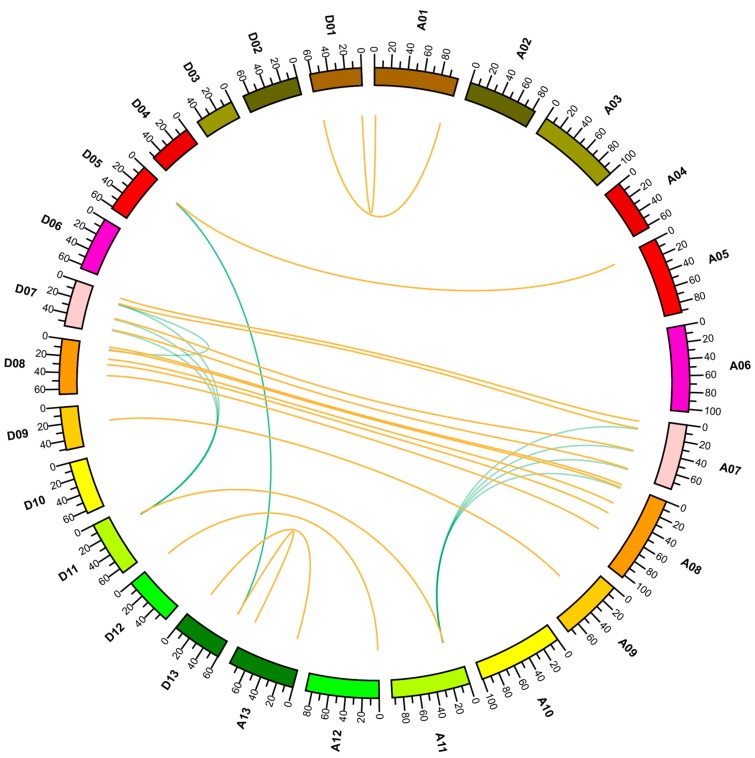
Collinearity analyses within At and Dt subgenomes. The orange lines indicate the ortholog genes from the At and Dt subgenomes. The green lines point towards paralog pairs derived from segmental duplication or whole genome duplication (WGD).

Because most of the orthologs on homologous chromosomes had significant collinearity between At and Dt subgenome, the gene expansion could have occurred before upland cotton formation. Almost half of the *CO-like* genes were identified as singletons, six genes might have originated from dispersed duplication, and 13 genes were found in collinear blocks, which may have undergone segmental duplication or whole genome duplication (WGD). Except for the *GhCOL17*–*GhCOL18* duplication pair, we noticed that the remaining collinear pairs were from Group III, indicating that segmental duplication or WGD was the major driver of the expansion of Group III. Among the duplicated genes, *GhCOL14_At* formed four pairs of genes with significant collinearity, and its ortholog *GhCOL14_Dt* formed three pairs of genes with significant collinearity, indicating that they were active during evolution. There were no tandem and proximal duplications in the chromosomal regions nearby or adjacent to the *CO-like* genes (Figure 2, Table S3).

Cotton plants have experienced many duplication events and the gene number has enlarged during evolution. The duplicated genes might have experienced functional divergence, including partial or total loss of the original functions, acquisition of novel functions, or maintenance of a partition of original functions [31]. The *CO-like* genes may have undergone positive selection, negative selection, and purifying selection during evolution. To inspect the selective pressures exerted on *CO-like* genes better, we evaluated the ratio of the non-synonymous divergence levels (Ka) versus synonymous divergence levels (Ks) of 27 homologous pairs, including 18 orthologous pairs and nine paralogous pairs derived from segmental duplication/WGD. All the values of Ka/Ks ratios were smaller than 1.0, among which the ratio for five gene pairs was >0.5 and those for five other gene pairs were <0.2 (Table 1), indicating that the *CO-like* gene family in cotton has experienced relaxed purifying selection pressure, and that their functions may be conserved after gene family expansion.

TEs are the major drivers of cotton genome expansion; particularly, the long-terminal repeat retrotransposons (LTR) is the major cause for size difference between the A genome (*G. arboreum*) and the D genome (*G. raimondii*) [28]. TEs are involved in gene family expansion, in addition to playing important roles in the regulation of gene expression under stress stimulus [32]. The 2000 bp region around the *CO-like* gene loci contained one DNA transposon and 12 retroelements (Table 2), including one PIF-Harbinger, four L1-type TEs, and eight copia-type TEs (Table 2 and Table S4). By enlarging the scanned region to 10 Kb, many TEs were identified, including that six DNA transposons and 51 retroelements were present. 

The DNA transposons were composed of four MULE-MuDR, one PIF-Harbinger, and one hAT-Charlie, whereas the retroelements were mainly composed of LTRs, including 36 copia-type and 11 Gypsy-type retroelements (Table 2 and Table S5). When the TE distribution was inspected further, we found three L1-type and four copia-type TEs located in the genomic region of *GhCOL12_Dt*, one PIF-Harbinger located in *GhCOL9_Dt*, three copia-type TEs located upstream of *GhCOL13_Dt*, and one L1 and four copia-type TEs inserted in the genomic region of *GhCOL12_Dt* within a 2000 bp region. Within the 10,000 bp region, we found TEs near the loci of 18 genes. For example, three copia TEs were distributed downstream of *GhCOL9_A*t, two copia-type TEs were located upstream of *GhCOL4_At*, and one copia-type TE was located downstream of *GhCOL4_At*. When comparing the origin of the TEs, we found more TEs from the At subgenome than from the Dt subgenome. Simple repeat sequences are more variable and widely spread in the whole genome across the upstream, downstream, and intragenic regions of *CO-like* genes than TEs are. *GhCOL13_At* and *GhCOL13_Dt* shared the same type of TEs belonging to ATCopia29, and *GhCOL12_At* and *GhCOL12_Dt* shared the same L1-type TE, indicating that these TEs are ancient and occur in the progenitor genomes of the At and Dt subgenomes.

### 2.5. The Effect of Domestication on CO-like Genes

**Figure 3:**
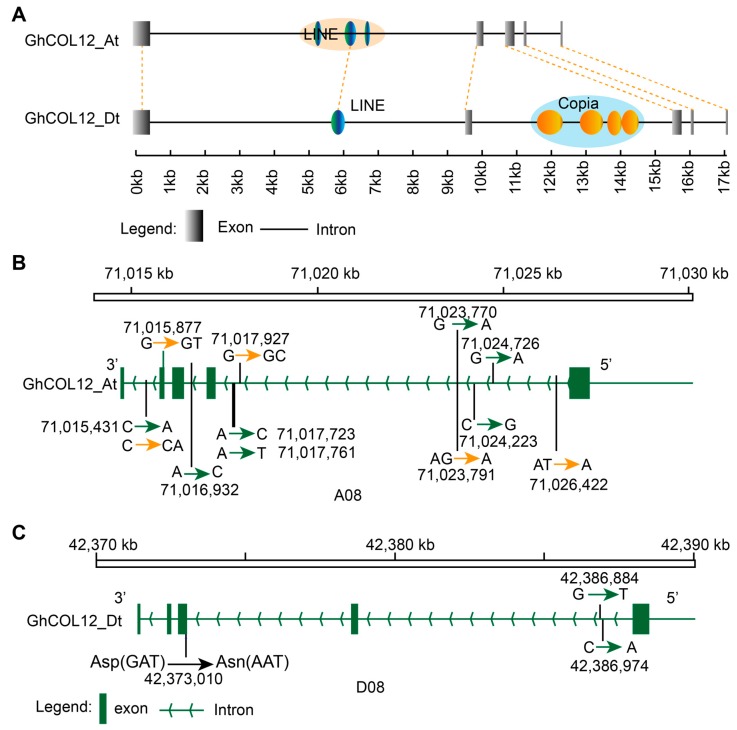
Domestication sweep analysis indicating that *GhCOL12_Dt* was a potential candidate under human-guided evolution: genomic character of *GhCOL12_At* and *GhCOL12_Dt* (**A**); genetic diversity of *GhCOL12_At* (**B**); and *GhCOL12_Dt* (**C**). The orange and green arrawhead lines indicated SNPs and Indels respectively. The digits closed to the arrawhead lines indicated the SNPs or Indels loci. The black vertical line indicated the SNPs or Indels loci on the chromosomes.

We checked whether the CO-like genes have experienced domestication or not by scanning the *CO-like* genes in the domestication sweep region and found that *GhCOL12* (*Gh_D08G1289*) was located in approximately 1.6 M region on D08 from 41,400,001–42,600,000 bp. Therefore, we compared the genomic sequences of *GhCOL12_At* and *GhCOL12_Dt*, and found that the exons were significantly conserved, whereas the introns were not. Three LINE-type TEs are located in the first intron of *GhCOL12_At*, whereas only one is located in *GhCOL12_Dt*. Four copia-type of TEs were inserted in the second intron of *GhCOL12_Dt*, but none in *GhCOL12_At* (Figure 3A). There were more sites with single nucleotide polymorphisms (SNPs) in the genomic region of *GhCOL12_At* than in *GhCOL12_Dt* (Figure 3B,C), indicating that *GhCOL12_At* maintained more diversity during evolution than did *GhCOL12_Dt*. We found seven SNPs and five indels in the *GhCOL12_At*, among which one indel was present in the fourth exon. As compared with *GhCOL12_At*, *GhCOL12_Dt* showed lower diversity in the genomic sequences because only three SNPs were detectable in the genomic region of *GhCOL12_Dt*. In the third exon of *GhCOL12_Dt*, a nonsynonymous mutation transformed Asp to Asn.

### 2.6. Gene Structure Analysis

**Figure 4:**
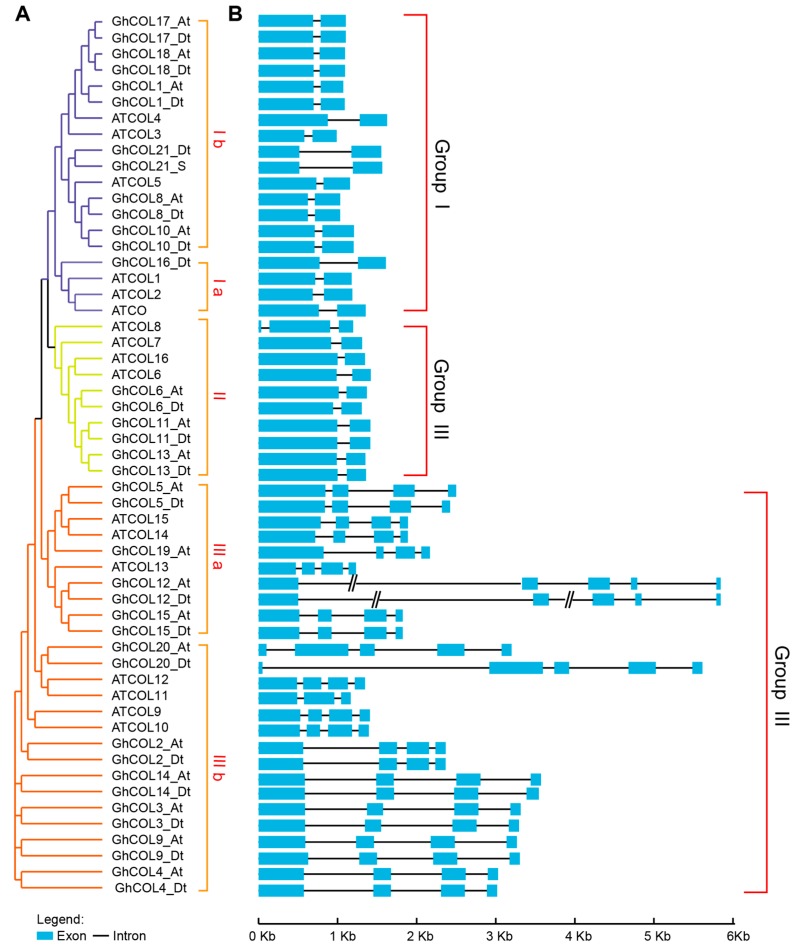
Comparison of the gene structures between *G. hirsutum* and *A. thaliana*. An NJ tree based on the relationship between *A. thaliana* and *G. hirsutum* (**A**); and the number, position, and length of exons and introns within CO-like genes (**B**). Purple, green, and orange in (**A)** represent Group I–III, respectively. Boxes and black lines in (**B**) indicate the exons and introns, respectively.

We found that the genes from cotton and *Arabidopsis* can be divided into three groups as those in Figure 1 (Figure 1 and Figure 4A). Because of the difference in number of genes, the topologies of the two trees displayed some general differences. However, the members within the same clade were the same and the topologies were similar. We found that *GhCOL14*, *GhCOL3*, *GhCOL4*, and *GhCOL9* clustered together, displaying a close phylogenetic relationship, which significantly supported the result shown in Figure 2. Figure 2 shows that Group III had undergone gene expansion. The genes shown in Figure 4A were inclined to cluster in cotton- or *Arabidopsis*-specific manner, suggesting that gene expansion occurred after divergence of cotton and *Arabidopsis*.

As shown in Figure 4B, *CO-like* genes contained multiple exons ranging from two to five. Although the number of exons differed among the groups, the exon patterns within groups were similar. For example, except *AtCOL8*, the remaining members in Group I and II contained two exons, and the first one was longer than the second one. Except *GhCOL12_At/Dt*, *GhCOL20_At/Dt*, and *AtCOL11*, the other genes of Group III were composed of four exons, among which the first one was the longest. Comparing gene structure of the orthologs from At and Dt subgenomes, the length of exons from the same position were found to be nearly equal, except for *GhCOL9_At/Dt*, *GhCOL16_At/Dt*, and *GhCOL20_At/Dt* gene pairs, indicating that the orthologs between At and Dt were conserved.

### 2.7. Gene-Expression Patterns in Different Tissues

Gene expression is the bridge between the genomic sequence and plant phenotype. Therefore, we inspected the CO-like gene-expression profiles in different tissues. As shown in Figure S4A, most of the genes were detectable in most of the tissues checked in our study, and we found that the expression levels differed between non-orthologous pairs, indicating that these genes may play diverse roles in different biological processes. As shown in Figure S4A, the genes were clustered according to their expression pattern, and of the genes with similar expression patterns in the heatmap displayed a more positive correlation. Based on the expression pattern, the genes could be roughly divided into a, b, and c clades. All the genes in clade a belonged to Group III, most of those in clade c belonged to Group III, and the genes in clade b were a mixture of the genes from different groups. Most of the genes were highly expressed in the torus and calycle, suggesting that they may be involved in regulation of flowering time. *GhCOL3_At/Dt*, *GhCOL1_A*t, and *GhCOL4_At* have relatively high expression level in the early stage of ovule development, suggesting they may be associated with ovule development. Only *GhCOL9_Dt* was highly expressed during the entire fibre elongation stage, and only six and two genes were highly expressed at 20 days-post-anthesis (DPA) and 5 DPA during the fibre development stage. Ten out of 19 pairs of orthologs showed a highly similar expression pattern, and the remaining orthologous pairs showed some differences, suggesting that these orthologous pairs might have undergone functional diversity. For example, *GhCOL12_At* and *GhCOL12_Dt* showed different expression patterns, whereas *GhCOL12_Dt* displayed a higher expression level than *GhCOL12_At* in stem and leaves. In addition, we checked the expression level of *CO-like* genes during seed development. As shown in Figure S4B, most of the genes expressed during seed development, and most of these expressed genes have a low expression levels during the early stage of seed germination. Moreover, the expression level of more than half of the genes showed a tendency of increased expression in the cotyledon. *GhCOL4_At*/*Dt*, *GhCOL21_At*/*Dt*, and *GhCOL5_At* showed higher expression levels than that of the other genes in roots.

### 2.8. Response of CO-like Genes to Photoperiod and Stress

**Figure 5:**
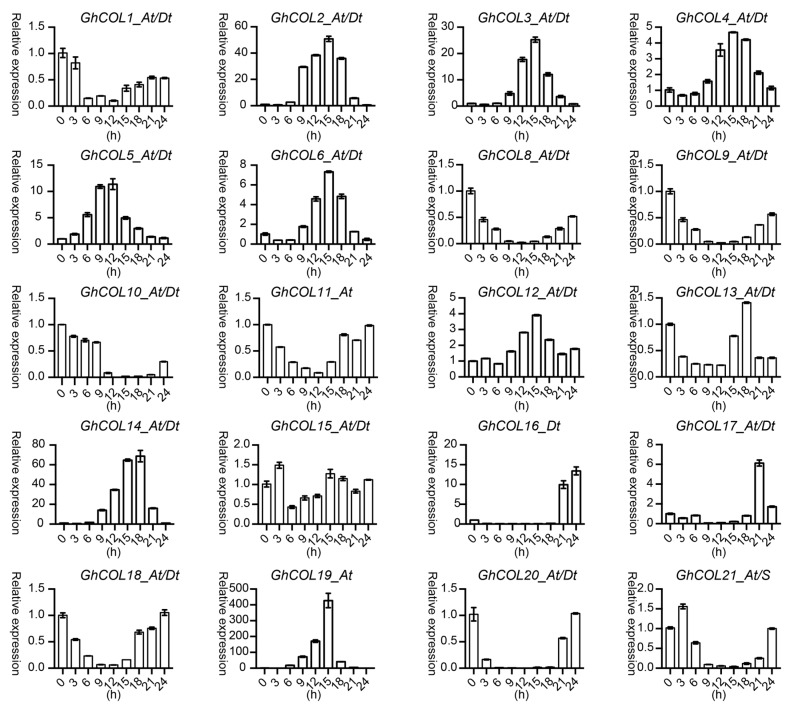
qPCR analysis showed that *CO-like* genes responded to the photoperiod. The cotton seedlings were sampled at different times over a 24 h period. The error bar represents the standard deviation of three biological replicates.

**Figure 6:**
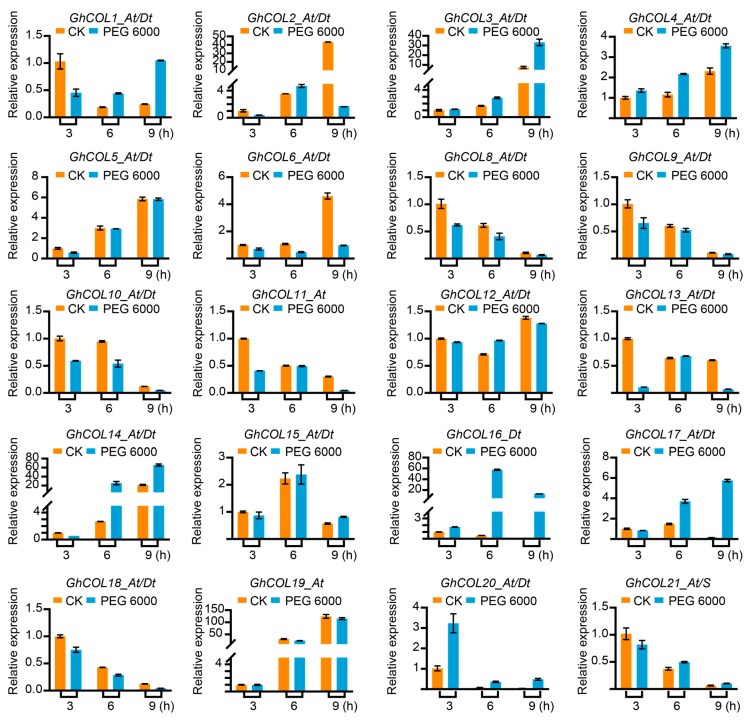
The qPCR analysis showed that *CO-like* genes might play important roles in drought stress. Four biological replicates were used in this study. The error bar represents the standard deviation of at four biological replicates.

**Figure 7:**
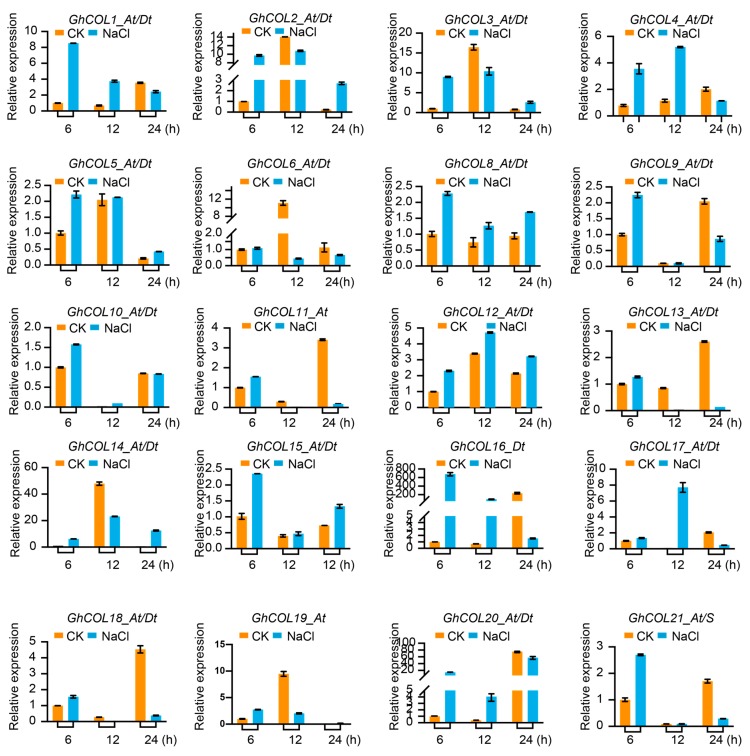
The qPCR analysis displayed that *CO-likes* may play important roles in salt stress. Four biological replicates were used in this study for qPCR. The error bar represents the standard deviation of at four biological replicates.

Figure 5 shows that the expression of most genes varied at times, indicating that the *CO-like* genes responded to the photoperiod. The expression pattern clustered into two groups: in the first, gene expression was initially up-regulated and then down-regulated, while the reverse occurred in the second group. As shown, *GhCOL2_At/Dt*, *GhCOL3_At/Dt*, and *GhCOL4_At/Dt* had relatively low expression levels at 0, 3, and 6 h, were significantly up-regulated from 9 h, peaked at 15 h, and then were down-regulated. However, the expression of *GhCOL8_At/Dt*, *GhCOL11_At/Dt*, and *GhCOL18_At/Dt* was down-regulated from 3 h, decreased to the lowest level at 12 h, and then was up-regulated thereafter. As shown in Figure 6, *CO-like* genes displayed diverse expression patterns under drought stress. *GhCOL3_At/Dt*, *GhCOL4_At/Dt*, *GhCOL14_At/Dt*, *GhCOL17_At/Dt*, *GhCOL16_At/Dt*, and GhCOL20_At/Dt were all up-regulated; the expression of the first eight genes peaked 9 h after treatment, while that of *GhCOL20_At/Dt* peaked 3 h after treatment. However, *GhCOL6_At/Dt*, *GhCOL8_At/Dt*, *GhCOL9_At/Dt*, *GhCOL10_At/Dt*, *GhCOL11_At/Dt*, *GhCOL13_At/Dt*, *GhCOL18_At/Dt*, *GhCOL19_At*, and GhCOL21_At/S were down-regulated when exposed to salt stress. By contrast, the expression of *GhCOL1_At/Dt*, *GhCOL2_At/Dt*, *GhCOL3_At/Dt*, *GhCOL4_At/Dt*, *GhCOL8_At/Dt*, *GhCOL12_At/Dt*, *GhCOL14_At/Dt*, *GhCOL16_At/Dt*, and *GhCOL17_At/Dt* was up-regulated under salt stress (Figure 7). *GhCOL3_At/Dt*, *GhCOL4_At/Dt*, *GhCOL14_At/Dt*, *GhCOL16_At/Dt*, and *GhCOL17_At/Dt* were up-regulated under both PEG 6000 and salt treatments, indicating that they may act as positive regulators during stress responses. *GhCOL6_At/Dt*, *GhCOL11_At/Dt*, *GhCOL13_At/D*t, and *GhCOL18_At/Dt* were down-regulated under both PEG 6000 and salt treatments, suggesting that they may be negative regulators. 

## 3. Discussion

### 3.1. CO-like Genes Have Experienced Expansion

*CO-like* genes encode a small number of transcriptional factors. In the present study, we found that the numbers of *CO-like* gene family members in each diploid species were different, and that cacao had the fewest genes, which was almost half of that in its close relative *G. raimondii*, suggesting that the *CO-like* genes in cotton have experienced expansion. A previous study showed that both cacao and cotton only experienced the paleohexaploidisation event shared by the eudicots, and that cotton has experienced recent duplication events but cacao not, which may be the major reason why the diploid cotton has more *CO-like* genes [26]. In this study, we found that the number of *CO-like* genes in *G. hirsutum* were almost twice of that in *G. arboretum* and *G. raimondii*, because *G. hirsutum* is the typical allotetraploid that was formed by the hybridisation of the A genome and the D genome, followed by chromosome doubling 1–2 million years ago [33]. *G. hirsutum* was very conserved after polyploidy because the orthologs from At and Dt genome maintained a significant collinearity with each other. 

In addition to the WGD, the segmental duplication was a major contributor to gene expansion in the CO-like gene family. Segmental duplication is very common in plants because most plants are diploidised polyploids and maintain large amounts of duplicated chromosomal blocks within their genomes [34]. In our previous study, we found that the segmental duplication was associated with WOX and YABBY genes expansion, and that the genes with segmental duplication showed different expression patterns [22,23]. In the present study, we found nine paralogous pairs were involved in segmental duplication or WGD; however, their expression patterns were similar with a very small value of Ka/Ks between paralogous gene pairs, indicating that these genes may be highly conserved during the evolution.

Cotton is the one of the most important crops in the world and it has been domesticated from the wild-type cotton plants under long-term human selection. Wild cotton species are perennial and are sensitive to photoperiod, whereas the cultivated cotton is photoperiod insensitive, which makes cotton widely cultivated in regions with different sunshine durations [30]. *CO-like* genes play significant roles in regulation of photoperiod pathway. The rice *Hd1*, an ortholog of *CO*, was a possible target of human selection during domestication of rice [35]. Wang et al. found 93 domestication sweeps occupying 178 Mb in the upland cotton genome by comparing the genetic diversity between cultivate cultivar and wild cotton species [36]. Based on comparison of the nucleotide diversity between the wild species and cultivars, we found that *GhCOL12_Dt* was a candidate gene for domestication selection. The genomic region of *GhCOL12_At* and *GhCOL12_Dt* contained different types of TEs and the *GhCOL12_At* displayed a higher diversity than *GhCOL12_Dt* did, because *GhCOL12_At* contained more SNPs than did *GhCOL12_Dt*. Furthermore, the expression pattern of *GhCOL12_D*t and *GhCOL12_At* differed in the ovule. Overall, it appeared that *GhCOL12_Dt* and *GhCOL12_At* might have experienced divergence, and that further experimental validation of this finding is required. 

TEs are mobile DNA elements, which are abundant in the genome and distributed in different positions [37]. TE activity and genomic recombination are the two major contributors to gene divergence and play important roles in species divergence. According to the dependence TE mobility on RNA, TEs are classified into retrotransposons and DNA transposons. Retrotransposons are the most abundant TEs in the cotton genome and were divided into LTR and non-LTR types [29]. In the present study, we found that the TEs around the *CO-like* genes are retrotransposons, and most of them are of the LTR type; moreover, the number of Copia-type TEs was larger than the Gypsy-type TEs, suggesting that Copia may be more active during the cotton evolution. TEs in At may be more active because the At subgenome has more TEs than Dt has. A previous study showed that the TEs may have burst in the progenitor genomes of upland cotton before its formation [30]. However, the distribution of TEs around genes from At and Dt differed, suggesting that these TEs might occur independently in the progenitors of At and Dt. *GhCOL13_At* and *GhCOL13_Dt* shared the same type of TEs belonging to ATCopia29, and *GhCOL12_At* and *GhCOL12_Dt* shared the same L1-type TE, indicating that these TEs are ancient and occur in the progenitor genomes of the At and Dt subgenomes.

### 3.2. CO-like Genes Were Highly Conserved during the Evolution

*CO-like* genes were characterised by the CCT and B-box domain. CCT domains are highly conserved among *CO-like* genes and B-box domains were more divergent in cotton (Figure 1), and this situation was also found in *Arabidopsis*, barley, and rice [1], suggesting that B-box may play significant roles in *CO-like* gene divergence. Although the sequences of B-boxes are diverse in different genes, they shared a higher conserved expression pattern as C-X2-C-X8-C-X7-C-X2-C-X4-H-X8-H, indicating that cysteine residues play important roles in maintaining the stability of the zinc-finger structure. A previous study showed that *CO-like* genes can be divided into three clades depending on the number and diversity of the B-box [1]. In the present study, we found that *CO-like* genes from cotton could be divided into three groups (Figure 1 and Figure S1). Members from the same groups showed similar characteristics. Firstly, the genes displayed more similarities in the amino sequences within groups (Figure S1). Secondly, the genes within groups showed a pattern; for example, the genes from Groups II and III mostly consisted of two exons and those from Group I mostly consisted of four exons (Figure 4). Finally, the genes within groups showed a similar expression pattern, particularly in Groups I and III (Figure S4). Most of the genes involved in duplication were from Group I and they displayed a similar expression pattern, indicating that the duplication was a major contributor to the expansion of the genes clustered in Group I, and that functional redundancy may exist among these genes. We found that each group was composed of genes from dicots and monocots, and the genes tended to clustered in dicot- or monocot-specific manner, indicating that the main function has been formed before dicot-monocot divergence.

### 3.3. CO-like Genes Play Diverse Roles under Stress

*CO-like* genes have been well studied for their roles in regulation of flowering time [9,10,11,12,13,14,15,16], but their roles in other biological process are poorly understood. *Ghd2* (*Os02g49880*), belonging to Group II, was significantly down-regulated in rice under drought stress [18], which corresponded to *GhCOL6_At/Dt*, *GhCOL11_At/Dt*, and *GhCOL13_At/Dt* in upland cotton. Intriguingly, *GhCOL6_At/Dt*, *GhCOL11_At*, and *GhCOL13_At/Dt* were differentially expressed under artificial drought stress, indicating that the genes from Group II might play significant roles in drought stress response. *AtCOL4* was up-regulated under salt, ABA, and osmotic stress, and its ortholog *GhCOL1_At/Dt* displayed an up-regulated expression pattern under both PEG 6000 and salt stress treatments, indicating that the functions of the genes were conserved during their evolution. Furthermore, *GhCOL18_At*/*Dt* displayed a close relationship with *GhCOL1_At/Dt*, which was down-regulated during the PEG treatment, suggesting that *GhCOL18_At/Dt* may act as a negative regulator under drought stress conditions. Most of the genes involved in duplication belonged to Group III b, among which *GhCOL2_At/Dt*, *GhCOL3_At/Dt*, and *GhCOL14_At/Dt* were differentially expressed under both drought and salt stress treatments, indicating that genes in Group III b may be involved in stress response and are candidate genes suitable for further study.

## 4. Materials and Methods

### 4.1. Gene Identification and Sequence Retrieval 

Cotton genome databases for the cotton plants *Gossypium arboreum* (BJI, version 1.0), *G. raimondii*, (JGI, version 2.0), and *G. hirsutum* (NAU, version 1.1) were available from COTTONGEN [38]. The genomic data of rice (version 7.0), sorghum (version 2.1), maize (version 1.1), and cacao (version 1.1) were downloaded from JGI (https://phytozome.jgi.doe.gov/pz/portal.html). The 17 amino acid sequences of *CO-like* genes from *Arabidopsis thaliana* were retrieved from The *Arabidopsis* Information Resource, version 10 (TAIR 10) (http://www.arabidopsis.org). *BLASTP* and *TBLASTN* programs were used to search the *G. raimondii* protein database using all protein sequences of *Arabidopsis* CO-like genes as queries, and a cut-off e-value of 1e^–5^. All candidates obtained from BLAST were extracted from the genome database for functional annotation. Interproscan 63.0 was used to annotate the CCT and B-box domain in the candidate sequences. *CO-like* genes from other species were analysed as described above for *G. raimondii*.

### 4.2. Conserved Sequence and Phylogenetic Analysis

For conserved domain analysis, the sequences were extracted from the full-length of CO-like protein sequences according to the domain positions retrieved from Interproscan 63.0, and ClustalX 2.0 was used for multiple-sequence alignments with the default parameters. For phylogenetic analysis, we used the ClustalW program (build-in MEGA 7.0) [39] to perform the multiple sequences alignment using the full-length sequences from rice, sorghum, maize, cacao, *Arabidopsis*, *G. arboreum*, *G. raimondii*, and *G. hirsutum*. Thereafter, we prepared a phylogenetic tree using the neighbour-joining (NJ) method with 1000 bootstrap replicates. Substitution was evaluated by the Poisson model using the default parameters. To validate the NJ tree, we used the Maximum likelihood method to construct an ML tree. Substitution was evaluated by the Poisson model, and bootstrap method (100 replications) was used to test the tree. A discrete Gamma distribution was used to model evolutionary rate differences among sites (5 categories (+G, parameter = 0.8524)). All positions with less than 95% site coverage were eliminated. 

### 4.3. Chromosomal Location and Collinearity Analysis

The exon loci were extracted using the method described in our previous study [22]. The chromosomal location was displayed by MapChart [40]. For collinearity analysis, the entire cotton protein sequences of upland cotton were aligned with each other using the basic local alignment search tool (BLAST) with a cut-off e-value of 1 × 10^−5^. The blastp result was analysed by MCScanx software to produce collinearity blocks across the whole genome. Collinearity pairs within the *CO-like f*amily of proteins were extracted to draw a collinearity map with CIRCOS (Version 69.0) software [41]. To analyse the duplicate gene type, the script duplicate_gene_classifier in MCScanx was used to identify the gene type, including singleton, dispersed duplicate, proximal, tandem, segmental duplication/whole genome duplication (WGD), using the blastp result as input with default parameters. 

### 4.4. Calculating Synonymous and Non-Synonymous Substitution Rates

Amino acid sequences from homologous pairs were aligned using Clustal X 2.0 (http://www.ebi.ac.uk/tools/clustalw2) with default parameters. The alignment results were converted to a codon alignment using the online tools PAL2NAL (http://www.bork.embl.de/pal2nal/). The CODEML program was used to calculate the synonymous (*Ks*) and non-synonymous (*Ka*) substitution substitution rates with default parameters [42].

### 4.5. Annotation and Analysis of Transposable Elements

The method for Transposable elements (TEs) were identified in upland cotton according to the method used in our previous study [22]. To analyse the distribution of TEs around *CO-like* genes, the TEs located 10,000 and 2000 bp upstream and downstream of the *CO-like* genes were analysed, and we performed statistical analysis of the different types of TEs present.

### 4.6. Gene Structure Analysis

We used the full-length *Arabidopsis* and *G. hirsutum* protein sequences to perform multiple alignments with ClustalW, and used MEGA 7.0 to construct an NJ tree using same method and parameters mentioned above. To draw the gene structure, we extracted the exon positions from the gff3 file using a Perl script, and used the online tool GSDS 2.0 for displaying it [43].

### 4.7. Transcriptome Data Analysis and Gene-Expression Heatmap

The raw data for RNA-Seq used in this study were downloaded from the national center for biotechnology information center (NCBI) sequence read archive (SRA: PRJNA248163) and analysed using the methods described in our previous study [22]. TopHat (Version 2.0.13) was used for short read mapping with default parameters, and cufflinks (Version 2.2.1) were used to calculate gene-expression levels with default parameters, and fragments per kilobase million (FPKM) values were used to normalise gene-expression levels [44]. The R package “ggplot2” was used to prepare the heatmap.

### 4.8. Real-Time PCR Analysis

The seeds of upland cotton TM-1 were obtained from the Cotton Research Institute of Chinese Academy of Agricultural Sciences.The seedlings were prepared as our previous report [22]. At the 3-leaf stage, the seedlings were treated with 300 mM, 10% PEG 6000 and water. To study the genes expression pattern in photoperiods, we sampled the roots at 0 (08:00), 3, 6, 9, 12,15,18, 21 and 24 h in normal growth condition (water treatment). For stress analysis, we sampled at 3, 6 and 9 h post PEG 6000 treatment and sampled at 6, 12 and 24 h post NaCl treatment. All samples were immediately frozen in liquid nitrogen and stored at −80 °C. The total RNA was extracted from the roots using RNAprep Pure Plant Kit (TIANGEN, Beijing, China). The first strand cDNA was synthesised using a PrimeScript® RT reagent kit (Takara, Dalian, China) was used to synthesise the first strand of cDNA, and SYBR Premix Ex TaqTM II was used in PCR amplifications. The house-keeping gene, Ghistone3 (GenBank accession no. AF024716), was used as an internal control, and the 2^−ΔΔ*C*t^ method was used to calculate the relative expression level.

## 5. Conclusions

Previous studies show that members of the *CO-like* gene family play important roles in the regulation flowering and stress response [9,10,11,12,13,14,15,16,17,18,19,20,21]. The results of the present study suggest that *CO-like* genes are significantly conserved among cotton and other plant species. Furthermore, whole genome and segmental duplications were two major contributors to the expansion of *CO-like* genes of cotton. *GhCOL12_Dt* is a candidate gene involved in selection by humans, which may be an important gene determining the insensitivity of cultivated cotton plants to photoperiod. Moreover, members from Group II and the duplicated genes in Group III might be involved in the mediation of stress responses. Our findings will not only deepen the understanding of the evolution of *CO-like* genes in cotton, but also facilitate further functional genomic studies on *CO-like* genes in cotton.

## Figures and Tables

**Table 1:** The *Ka* and *Ks* value of homologous gene pairs.

Paralogous Pairs	Ka	Ks	Ka/Ks
*GhCOL1_At-GhCOL1_Dt*	0.03	0.07	0.526
*GhCOL2_At-GhCOL2_Dt*	0.00	0.02	0.149
*GhCOL3_At-GhCOL3_Dt*	0.01	0.04	0.199
*GhCOL4_At-GhCOL4_Dt*	0.01	0.05	0.275
*GhCOL5_At-GhCOL5_Dt*	0.01	0.04	0.365
*GhCOL6_At_GhCOL6_Dt*	0.01	0.03	0.550
*GhCOL8_At_GhCOL8_Dt*	0.01	0.04	0.298
*GhCOL9_At_GhCOL9_Dt*	0.00	0.06	0.054
*GhCOL10_At-GhCOL10_Dt*	0.01	0.06	0.159
*GhCOL11_At-GhCOL11_Dt*	0.03	0.05	0.561
*GhCOL12_At-GhCOL12_Dt*	0.01	0.02	0.552
*GhCOL13_At-GhCOL13_Dt*	0.01	0.03	0.365
*GhCOL14_At-GhCOL14_Dt*	0.01	0.06	0.131
*GhCOL15_At-GhCOL15_Dt*	0.01	0.04	0.276
*GhCOL17_At-GhCOL17_Dt*	0.01	0.03	0.205
*GhCOL18_At-GhCOL18_Dt*	0.01	0.05	0.274
*GhCOL20_At-GhCOL20_Dt*	0.04	0.05	0.993
*GhCOL21_S-GhCOL21_Dt*	0.04	0.07	0.515
*GhCOL3_Dt-GhCOL14_Dt*	0.09	0.41	0.213
*GhCOL4_Dt-GhCOL14_Dt*	0.09	0.40	0.223
*GhCOL9_Dt-GhCOL2_Dt*	0.11	0.53	0.211
*GhC0L18_Dt-GhCOL17_Dt*	0.09	0.73	0.119
*GhCOL2_Dt-GhCOL14_Dt*	0.09	0.53	0.175
*GhCOL3_At-GhCOL14_At*	0.08	0.41	0.204
*GhCOL2_At-GhCOL14_At*	0.09	0.45	0.210
*GhCOL9_At-GhCOL14_At*	0.09	0.45	0.210
*GhCOL4_At-GhCOL14_At*	0.09	0.39	0.230

**Table 2:** Transposable elements around the *CO-like* gene loci.

Type	Number of Elements	Length Occupied (bp)	Percentage of Sequence (%)	Number of Elements	Length Occupied (bp)	Percentage of Sequence (%)
	2000 bp region			10,000 bp region		
DNA transponsons	1	283	0.1	6	109	0.04
CMC-EnSpm	0	0	0	4	1172	0.42
MULE-MuDR	0	0	0	0	0	0
PIF-Harbinger	1	283	0.1	1	283	0.1
TcMar-Pogo	0	0	0	0	0	0
hAT	0	0	0	0	0	0
hAT-Ac	0	0	0	0	0	0
hAT-Tag1	0	0	0	0	0	0
hAT-Tip100	0	0	0	0	0	0
hAT-Charlie	0	0	0	1	32	0.01
Retroelements	20	6199	2.23	51	25,761	9.27
LINE:	0	0	0	0	0	0
L1	4	1085	0.39	4	1085	0.39
LTR:	8	2557	0.92	0	0	0
Caulimovirus	0	0	0	0	0	0
Copia	8	2557	0.92	36	19,874	7.15
Gypsy	0	0	0	11	4802	1.73
RC:	0	0	0	0	0	0
Helitron	0	0	0	0	0	0
Low_complexity	36	1880	0.68	117	6419	2.31
Simple_repeat	144	6167	2.22	481	20,564	7.4
Unspecified	24	3581	1.29	113	38,583	13.88

## Supplementary Materials

Supplementary Materials can be found at http://www.mdpi.com/1422-0067/19/9/2658/s1.
