# Supplementary material for: Genome-Wide Analysis Elucidates the Role of CONSTANS-like Genes in Stress Responses of Cotton
Source: Int J Mol Sci. 2018 Sep 7;19(9):2658. doi: 10.3390/ijms19092658 (PMC6165416; doi:10.3390/ijms19092658)
Supplement: Supplementary file 1 [file ijms-19-02658-s001.zip › Table S2.pdf]

Table S2: Gene loci information of cotton CO-like genes

Gene ID	Gene locus	Chr.	Direction	Start	End	Gene ID	Gene locus	Chr.	Direction	Start	End
GrCOL1	Gorai.001G039500.1	Chr01	-	3676945	3678125	GhCOL1_Dt	Gh_D07G0334	D07	-	3531553	3532642
GrCOL2	Gorai.001G099700.1	Chr01	-	11189054	11193404	GhCOL2_Dt	Gh_D07G0867	D07	-	11252477	11254842
GrCOL3	Gorai.001G190000.1	Chr01	+	31160664	31165694	GhCOL3_Dt	Gh_D07G1645	D07	+	32928032	32931325
GrCOL4	Gorai.001G223800.1	Chr01	-	45684954	45689894	GhCOL4_Dt	Gh_D07G1957	D07	-	48829548	48832565
GrCOL5	Gorai.002G022300.1	Chr02	+	1556020	1559124	GhCOL5_Dt	Gh_D01G0200	D01	+	1674672	1677093
GrCOL6	Gorai.002G218400.1	Chr02	-	57033477	57035219	GhCOL6_Dt	Gh_D01G1811	D01	+	55566742	55568047
GrCOL7	Gorai.003G104600.1	Chr03	-	32248407	32249974	NA	NA	NA	NA	NA	NA
GrCOL8	Gorai.004G030300.1	Chr04	-	2397135	2398635	GhCOL8_Dt	Gh_D08G0269	D08	-	2563414	2564444
GrCOL9	Gorai.004G061000.1	Chr04	+	6015304	6020382	GhCOL9_Dt	Gh_D08G0537	D08	+	6121175	6124445
GrCOL10	Gorai.004G102000.1	Chr04	+	18922045	18923759	GhCOL10_Dt	Gh_D08G0923	D08	+	19535668	19536870
GrCOL11	Gorai.004G113000.1	Chr04	+	25961240	25963398	GhCOL11_Dt	Gh_D08G1030	D08	-	27169749	27171161
GrCOL12	Gorai.004G140600.1	Chr04	-	39574008	39590545	GhCOL12_Dt	Gh_D08G1289	D08	-	42371421	42388486
GrCOL13	Gorai.006G061600.1	Chr06	+	22449476	22451500	GhCOL13_Dt	Gh_D09G0473	D09	-	23131764	23133122
GrCOL14	Gorai.007G164600.1	Chr07	+	14483656	14489122	GhCOL14_Dt	Gh_D11G1518	D11	+	15171039	15174582
GrCOL15	Gorai.008G008400.1	Chr08	-	1001595	1004408	GhCOL15_Dt	Gh_D12G0077	D12	-	1018268	1020091
GrCOL16	Gorai.008G059900.1	Chr08	+	9451492	9453391	GhCOL16_Dt	Gh_D12G0543	D12	+	9963089	9964699
GrCOL17	Gorai.009G065600.1	Chr09	-	4656585	4658271	GhCOL17_Dt	Gh_D05G0635	D05	-	5090553	5091655
GrCOL18	Gorai.010G245200.1	Chr10	-	61425655	61427048	GhCOL18_Dt	Gh_D13G1939	D13	+	54761440	54762533
GrCOL19	Gorai.012G083100.1	Chr12	+	13849379	13851855	NA	NA	NA	NA	NA	NA
GrCOL20	Gorai.013G071300.1	Chr13	+	8325283	8330097	GhCOL20_Dt	Gh_D13G0627	D13	+	8772549	8778161
GrCOL21	Gorai.013G246000.1	Chr13	-	56481092	56483308	GhCOL21_Dt	Gh_D13G2210	D13	-	58226829	58228380
GaCOL1	Cotton_A_19228	Ca1	+	49125900	49127163	GhCOL1_At	Gh_A07G0278	A07	-	3415559	3416629
GaCOL2	Cotton_A_26963	Ca1	-	51719221	51724440	GhCOL2_At	Gh_A07G0806	A07	-	13791448	13793814
GaCOL3	Cotton_A_36596	Ca1	+	50947820	50956549	GhCOL3_At	Gh_A07G1493	A07	+	45111751	45115065
GaCOL4	Cotton_A_31698	Ca1	-	115728056	115732856	GhCOL4_At	Gh_A07G1753	A07	-	71671991	71675019
GaCOL5	Cotton_A_00045	Ca4	-	37551220	37554155	GhCOL5_At	Gh_A01G0157	A01	+	1485300	1487798
GaCOL6	Cotton_A_27017	Ca1	-	109563005	109564356	GhCOL6_At	Gh_A01G1562	A01	-	92459249	92460618
GaCOL8	Cotton_A_06763	Ca7	-	52352678	52354110	GhCOL8_At	Gh_A08G0192	A08	-	1929796	1930828
GaCOL9	Cotton_A_03265	Ca7	+	27567756	27572884	GhCOL9_At	Gh_A08G0451	A08	+	6093346	6096612
GaCOL10	Cotton_A_34286	Ca6	+	32893404	32894609	GhCOL10_At	Gh_A08G0775	A08	+	29816347	29817552
GaCOL11	Cotton_A_35756	Ca12	+	87836722	87838138	GhCOL11_At	Gh_A08G0848	A08	+	45369135	45370548
GaCOL12	Cotton_A_34661	Ca7	-	39152793	39173051	GhCOL12_At	Gh_A08G1015	A08	-	71014737	71027302
GaCOL13	Cotton_A_38065	Ca10	+	61125170	61126742	GhCOL13_At	Gh_A09G0466	A09	+	36932150	36933501
GaCOL14	Cotton_A_03720	Ca3	+	52568546	52572120	GhCOL14_At	Gh_A11G1370	A11	+	17725612	17729183
NA	NA	NA	NA	NA	NA	GhCOL15_At	Gh_A12G0062	A12	-	892319	894142
GaCOL17	Cotton_A_01142	Ca3	-	33648414	33649978	GhCOL17_At	Gh_A05G0516	A05	-	5520316	5521418
GaCOL18	Cotton_A_09109	Ca5	-	49086069	49085991	GhCOL18_At	Gh_A13G1580	A13	+	74644413	74645506
GaCOL19	Cotton_A_29042	Ca2	-	55770739	55772849	GhCOL19_Dt	Gh_A05G2921	A05	+	70782683	70784849
GaCOL20	Cotton_A_28699	Ca5	+	60903023	60905668	GhCOL20_At	Gh_A13G0504	A13	-	11597181	11600380
GaCOL21	Cotton_A_02515	Ca6	+	51443246	51445174	GhCOL21S	Gh_Sca004822G01	Scaffold4822	+	3441	5005
