# Supplementary material for: Genome-Wide Analysis Elucidates the Role of CONSTANS-like Genes in Stress Responses of Cotton
Source: Int J Mol Sci. 2018 Sep 7;19(9):2658. doi: 10.3390/ijms19092658 (PMC6165416; doi:10.3390/ijms19092658)
Supplement: Supplementary file 1 [file ijms-19-02658-s001.zip › Table S3.docx]

Table S3: Duplication type information of CO-like genes

| Gene ID | Gene locus | Gene  type | Gene ID | Gene locus | Gene  type |
| --- | --- | --- | --- | --- | --- |
| GhCOL1_At | Gh_A07G0278 | 0 | GhCOL3_Dt | Gh_D07G1645 | 4 |
| GhCOL2_At | Gh_A07G0806 | 4 | GhCOL4_Dt | Gh_D07G1957 | 4 |
| GhCOL3_At | Gh_A07G1493 | 4 | GhCOL5_Dt | Gh_D01G0200 | 0 |
| GhCOL4_At | Gh_A07G1753 | 4 | GhCOL6_At | Gh_A01G1562 | 0 |
| GhCOL5_At | Gh_A01G0157 | 4 | GhCOL8_Dt | Gh_D08G0269 | 0 |
| GhCOL6_Dt | Gh_D01G1811 | 0 | GhCOL9_Dt | Gh_D08G0537 | 4 |
| GhCOL8_At | Gh_A08G0192 | 1 | GhCOL10_Dt | Gh_D08G0923 | 0 |
| GhCOL9_At | Gh_A08G0451 | 4 | GhCOL11_Dt | Gh_D08G1030 | 0 |
| GhCOL10_At | Gh_A08G0775 | 1 | GhCOL12_Dt | Gh_D08G1289 | 0 |
| GhCOL11_At | Gh_A08G0848 | 1 | GhCOL13_Dt | Gh_D09G0473 | 0 |
| GhCOL12_At | Gh_A08G1015 | 0 | GhCOL14_Dt | Gh_D11G1518 | 4 |
| GhCOL13_At | Gh_A09G0466 | 1 | GhCOL15_Dt | Gh_D12G0077 | 0 |
| GhCOL14_At | Gh_A11G1370 | 4 | GhCOL16_Dt | Gh_D12G0543 | 0 |
| GhCOL15_At | Gh_A12G0062 | 0 | GhCOL17_Dt | Gh_D05G0635 | 4 |
| GhCOL17_At | Gh_A05G0516 | 1 | GhCOL18_Dt | Gh_D13G1939 | 4 |
| GhCOL18_At | Gh_A13G1580 | 1 | GhCOL19_At | Gh_A05G2921 | 0 |
| GhCOL20_At | Gh_A13G0504 | 0 | GhCOL20_Dt | Gh_D13G0627 | 0 |
| GhCOL1_Dt | Gh_D07G0334 | 0 | GhCOL21_Dt | Gh_D13G2210 | 0 |
| GhCOL2_Dt | Gh_D07G0867 | 4 |  |  |  |

0, 1, 2, 3, 4 stand for singleton, dispersed, proximal, tandem, segmental.
