# Supplementary figures and images for: Genome-Wide Analysis Elucidates the Role of CONSTANS-like Genes in Stress Responses of Cotton
Source: Int J Mol Sci. 2018 Sep 7;19(9):2658. doi: 10.3390/ijms19092658 (PMC6165416; doi:10.3390/ijms19092658)

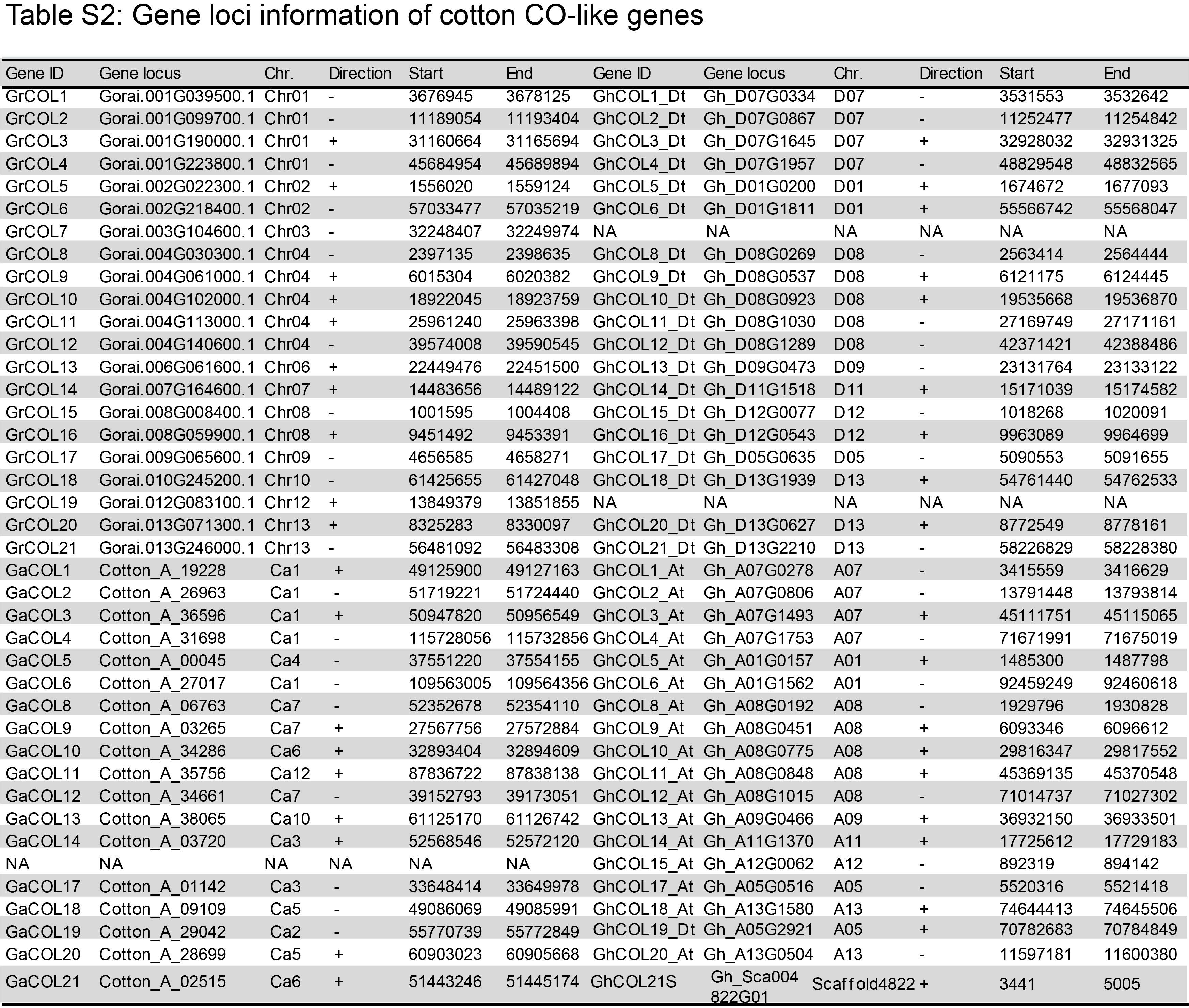

Supplement: Supplementary file 1 [file ijms-19-02658-s001.zip › Table S2.png]

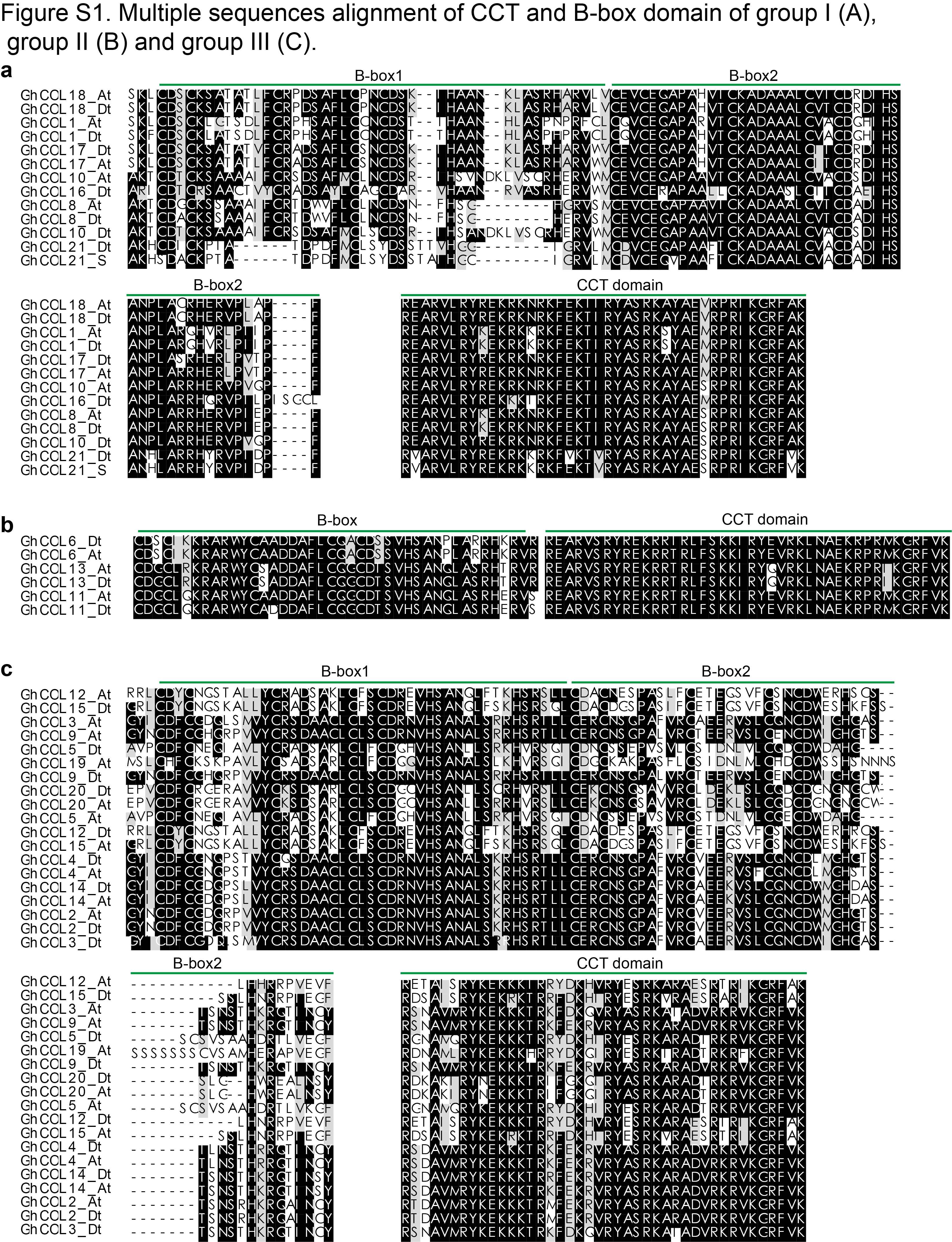

Supplement: Supplementary file 1 [file ijms-19-02658-s001.zip › Figure S1 .png]

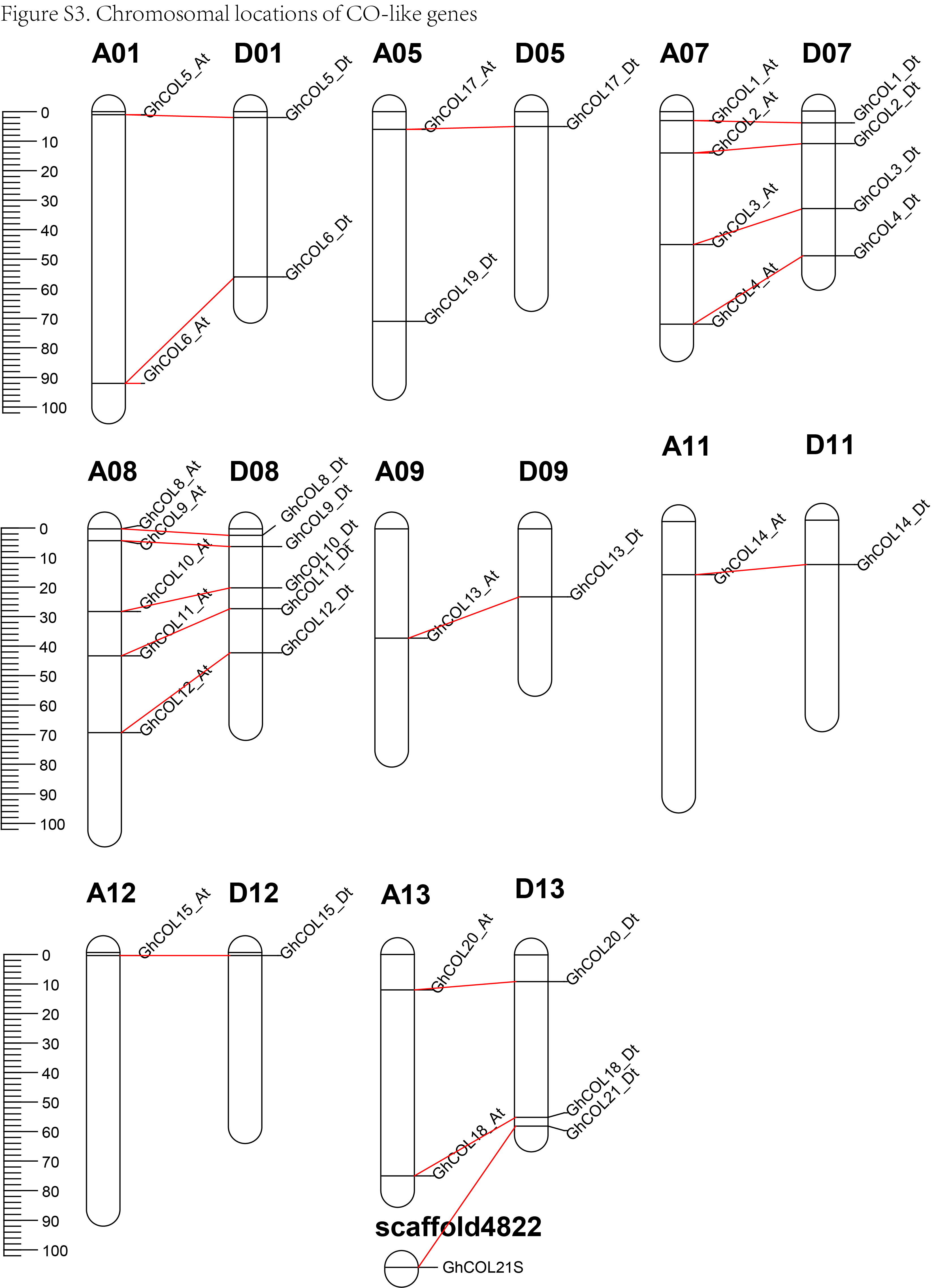

Supplement: Supplementary file 1 [file ijms-19-02658-s001.zip › Figure S3.png]

Group III

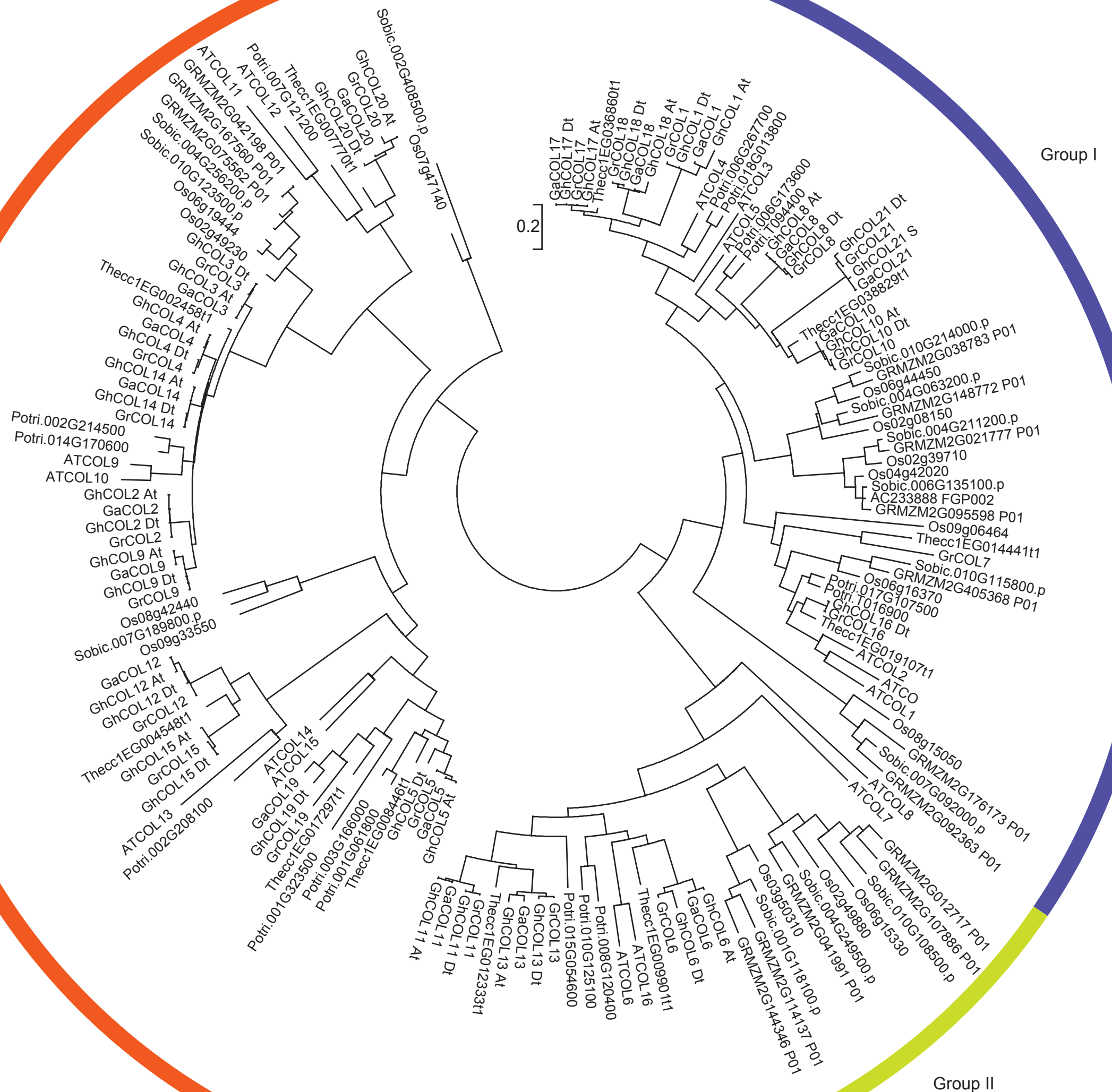

Supplement: Supplementary file 1 [file ijms-19-02658-s001.zip › Figure S2.pdf]

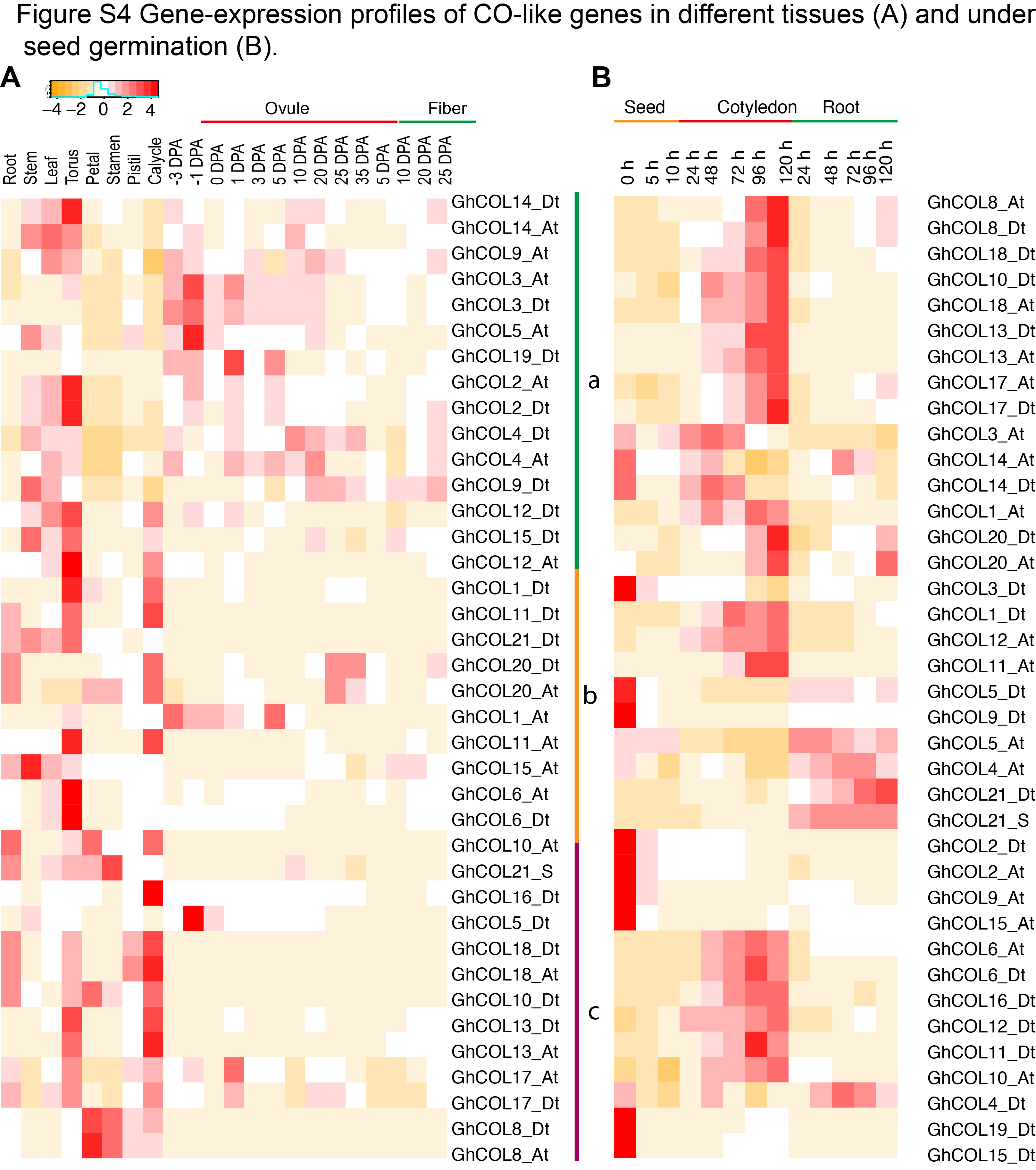

Supplement: Supplementary file 1 [file ijms-19-02658-s001.zip › Figure S4.png]
